# Efeito da Redução do Sal de Adição sobre a Pressão Arterial Central e Periférica

**DOI:** 10.36660/abc.20180426

**Published:** 2020-04-06

**Authors:** Ana Carolina Arantes, Ana Luiza Lima Sousa, Priscila Valverde de O. Vitorino, Paulo Cesar B. Veiga Jardim, Thiago de Souza Veiga Jardim, Jeeziane Marcelino Rezende, Ellen de Souza Lelis, Rafaela Bernardes Rodrigues, Antonio Coca, Weimar Kunz Sebba Barroso

**Affiliations:** 1 Universidade Federal de GoiásFaculdade de MedicinaPrograma de Pós-Graduação em Ciências da SaúdeGoiâniaGOBrasilUniversidade Federal de Goiás - Faculdade de Medicina - Programa de Pós-Graduação em Ciências da Saúde, Goiânia, GO – Brasil; 2 Universidade Federal de GoiásGoiâniaGOBrasilUniversidade Federal de Goiás - Liga de Hipertensão Arterial, Goiânia, GO – Brasil; 3 Pontificia Universidade Católica de GoiásEscola de Ciências Sociais e da SaúdeGoiâniaGoiásBrasilPontificia Universidade Católica de Goiás - Escola de Ciências Sociais e da Saúde - Mestrado em Atenção à Saúde, Goiânia, Goiás – Brasil; 4 Universitat de BarcelonaBarcelonaEspanhaUniversitat de Barcelona, Barcelona – Espanha

**Keywords:** Doenças Cardiovasculares, Pressão Arterial, Pré-Hipertensão, Hipertensão, Cloreto de Sódio, Dieta Hipossódica, Políticas de Saúde

## Abstract

**Fundamento:**

Os efeitos da redução na ingestão do sal sobre a pressão arterial (PA) casual de hipertensos já foram amplamente estudados, entretanto essa análise ainda é escassa no contexto da redução exclusiva do sal de adição na rigidez arterial e em indivíduos normotensos e pré-hipertensos.

**Objetivo:**

Avaliar os efeitos da redução progressiva na ingestão do sal de adição (de 6 para 4 g/dia) sobre os valores da pressão periférica e central, a rigidez arterial em normotensos, pré-hipertensos e hipertensos.

**Métodos:**

Ensaio clínico, simples cego com 13 semanas de seguimento. Foram avaliados normotensos (≤130/85 mmHg), pré-hipertensos (≥130 e <139/≥85 e <90 mmHg) e hipertensos estágio 1 (≥140 e <160/≥90 e <100 mmHg). Utilizou-se medida casual e monitorização residencial da PA com aparelho automático OMRON 705CP, medida central da PA com Sphygmocor^®^, dosagem do sódio urinário de 24h (colhido no intervalo entre cada visita) e mensuração de sal de adição. Foi adotado nível de significância p<0,05 para todas as análises.

**Resultados:**

Foram avaliados 55 participantes (18 normotensos; 15 pré-hipertensos; 22 hipertensos) com mediana 48 anos (IQ:39-54). Os grupos foram semelhantes em relação a idade e sexo. Não houve diferença entre medidas de PA e excreção de sódio antes e depois da intervenção. Os parâmetros de rigidez arterial também não sofreram alterações significativas.

**Conclusão:**

A redução gradativa da ingestão de sal de adição num seguimento de 13 semanas não foi capaz de reduzir de maneira significativa os valores periféricos e centrais da PA. (Arq Bras Cardiol. 2020; 114(3):554-561)

## Introdução

A hipertensão arterial sistêmica (HAS) é um dos fatores de risco cardiovasculares mais prevalentes. Atinge cerca de 970 milhões de indivíduos em todo o mundo. É a causa, direta ou indireta, de mais de 9 milhões de óbitos todos os anos,^1^ responsável por 62% dos casos de doenças cardiovasculares (DCV) e 49% de doença cardíaca isquêmica.^2^ A pré-hipertensão (PH) também está associada com aumento na incidência das DCV.^3,4^

Dentre as ferramentas capazes de avaliar a pressão arterial (PA) a medida casual, comparada com os outros métodos, apresenta inferioridade na predição de risco cardiovascular e na acurácia diagnóstica.^5,6^ A monitorização residencial da pressão arterial (MRPA) apresenta excelente acurácia diagnóstica e relação custo benefício.^7,8^ Já a medida central da pressão arterial (PAC), por avaliar a PA em grandes artérias, mais elásticas, apresenta valores mais baixos que a pressão casual e melhor associação com as lesões em órgãos alvo. É portanto, melhor preditora de eventos cardiovasculares^8^ e permite analisar parâmetros de rigidez arterial e resistência vascular.^9-12^

A etiologia do aumento da PA é multifatorial, entretanto a ingestão excessiva de sal é frequente e importante. Ocasiona aumento dos níveis pressóricos e complicações cardiovasculares. Desta forma, a restrição de sal é uma ferramenta importante para a prevenção e o controle da HAS e das DCV.^13,14^

A média recomendada de consumo é de 5 g de sal/dia/pessoa, ou < 2 g de sódio/dia/pessoa. Todavia, o consumo médio diário de sal dos brasileiros está acima do recomendado, alcançando até 12g/dia.^15^ Hoje, vários países definiram como política de governo a redução de pelo menos 30% na ingestão média de sal da população até o ano 2025 com o objetivo de diminuir os valores de PA.^16^

A importância em se avaliar o consumo do sal, estimular a sua restrição na dieta e utilizar ferramentas capazes de identificar essa redução é importante na estratégia de prevenção primária das DCV.

Dessa forma, este artigo avaliou o efeito da redução na ingestão do sal de adição na PA central e periférica em normotensos, pré-hipertensos e hipertensos após 13 semanas.

## Métodos

Trata-se de um sub-estudo realizado a partir da fase II do ensaio clínico, simples cego, controlado com diferentes dosagens de ingestão de sal de adição em grupos categorizados segundo a PA. Para a amostra inicial foram recrutados, no próprio local de trabalho, 1.000 servidores de uma universidade pública brasileira. Em toda essa amostra foram aplicados questionários sobre hábitos alimentares, realizadas medidas antropométricas e casual da PA. Após o recrutamento foram identificados 678 servidores que aceitaram participar da fase II do estudo (Figura 1).

**Figura 01:**
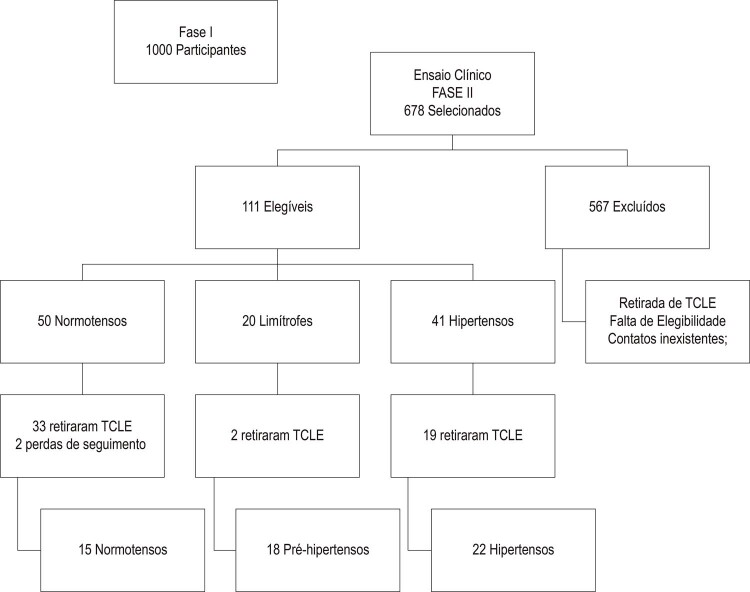
– *Fluxograma Fase II do Ensaio clínico. TCLE: termo de consentimento livre e esclarecido.*

O estudo foi aprovado pelo Comitê de Ética em Pesquisa da Instituição, Protocolo CAEE: 00790712.3.0000.5078 e todos os participantes assinaram o Termo de Consentimento Livre e Esclarecido antes de qualquer procedimento do estudo.

A amostra foi de conveniência e incluiu participantes com idade entre 20 a 60 anos, ambos os sexos e que, realizavam no mínimo quatro refeições principais (almoço e/ou jantar) por semana preparadas em seus domicílios.

Foram excluídos os participantes com PA casual ≥ 160/100 mmHg, diabéticos, com história de doença crônica e hipertensos em uso de dois ou mais anti-hipertensivos.

A participação no estudo consistiu na realização de 5 visitas com intervalo de 30±7 dias entre elas. A visita inicial foi dividida em dois momentos: Visita 1A (V1A) e Visita 1B (V1B). Em todas as visitas, foram realizadas: medida casual da PA, PAC, MRPA, medidas antropométricas, cálculo do índice de massa corporal (IMC), solicitação de urina tipo I (EAS), creatinina urinária e sódio e potássio urinário de 24 horas. Na V1A, além dos procedimentos mencionados os participantes assinaram o termo de consentimento livre e esclarecido, foram avaliados quanto aos critérios de elegibilidade e foi solicitado o exame de creatinina sérica.

Na V1B os grupos foram divididos, de acordo com a média da medida casual, em grupo normotenso (GN) (PA < 130/85 mmHg), grupo pré-hipertenso (GPH) (≥130 < 140/≥85 < 90 mmHg) e grupo hipertenso estágio 1 (GH) (≥140 e <160/≥ 90 e < 100 mmHg) sem uso de medicação ou qualquer valor de PA com uso de medicação anti-hipertensiva.^1^

Para a medida casual foram realizadas três medidas da PA com intervalo mínimo de um minuto entre elas. Quando havia diferença maior que 4 mmHg entre as medidas foram realizadas medidas complementares até que fossem obtidos valores com diferença inferior. Para fins de análise foi utilizada a média das duas últimas medidas.

Tanto para a realização da medida casual da PA, quanto para a MRPA, foram utilizados aparelhos semi-automáticos da marca OMRON, modelo HEM-711 ACINT, com braçadeiras adequadas à circunferência do braço. A PA foi aferida na posição sentada, após o repouso do indivíduo de pelo menos cinco minutos, em ambiente calmo, no braço com maior valor da PA.^1^

A medida de MRPA foi realizada de acordo com o protocolo estabelecido pelas III Diretrizes Brasileiras de Monitorização Residencial da Pressão Arterial.^17^ O aparelho de MRPA foi entregue aos participantes em todas as visitas. Eles foram orientados a realizar as medidas da PA conforme protocolo, anotar os valores em ficha específica para esse fim e devolver o aparelho no retorno agendado para a visita subsequente.

Para a medida da PAC foi utilizado o método de tonometria de aplanação com aparelho Sphygmocor^®^, calibrado e validado.^12^ O paciente permaneceu em repouso por cinco minutos, sem ter ingerido bebida alcoólica, café e nem ter fumado nas horas que antecedem o exame e também deveria estar com a bexiga vazia. As variáveis avaliadas a partir da medida da PAC foram: pressão arterial sistólica central (PASc), pressão arterial diastólica central (PADc), pressão de pulso central (PPc) e augmentation índex (AIx).

A metodologia para a coleta de urina de 24 horas foi orientada por meio de folheto explicativo e realizada no laboratório de análises clínicas da Universidade Federal de Goiás com a técnica membrana-íon-seletiva para quantificar o sódio urinário no início, antes da intervenção do estudo, e nos intervalos entre todas as visitas totalizando quatro coletas.

Na V1B os grupos, GN, GPH e GH receberam a mesma orientação sobre ingestão do sal de adição e 6g/dia/pessoa. Na Visita 2 (V2) e visita 3 (V3) foram fornecidos 5 e 4g/dia/pessoa, respectivamente. Os intervalos entre essas visitas foi de 30 ± 7 dias.

O quantitativo sal para o uso diário foi calculado de acordo com o número de moradores em cada residência considerando o preparo das refeições no almoço e jantar e, dispensados devidamente embalados e sem identificação do peso. Também foram dispensados 10% a mais da quantidade de sal calculada, para utilização em casos excepcionais, como por exemplo em dias com maior quantidade de pessoas em casa (visitas).

Nos retornos da V2, V3 e visita 4 (V4) foram recolhidas as embalagens de sal utilizadas e dispensadas novas embalagens com a quantidade de sal programada para o período subsequente, além das coletas dos exames programados. Em todas as visitas eram enfatizadas as orientações sobre a importância, para a saúde cardiovascular, da ingestão de alimentos com pouco sódio e que, o consumo de sal de adição nos dias programados fosse apenas aquele dispensado pelo protocolo.

As embalagens devolvidas (vazias e cheias) foram pesadas e utilizadas para a análise de adesão ao protocolo. Também foi utilizado para esse fim a análise da excreção de sódio urinário de 24 horas.

### Análise estatística

Os dados foram analisados com o programa Stata, versão 12. A análise estatística foi realizada por intenção de tratar, desta forma para os participantes que abandonaram o estudo antes da visita 4 foram considerados os dados da última visita realizada. As variáveis contínuas com distribuição normal foram apresentadas com média e desvio-padrão e aquelas com distribuição não normal, através de mediana e intervalos interquartis. Já as variáveis categóricas, foram apresentadas com frequência absoluta e relativa. Para verificar a distribuição dos dados das variáveis foi utilizado o teste de Shapiro-Wilk.

Para as comparações entre os grupos na V1A utilizou-se o Teste de Kruskal-Wallis e o Teste Exato de Fisher. Para as comparações intra grupo antes (V1B) e após (V4) a intervenção foram aplicados os testes de Wilcoxon ou teste t-Student pareado. Para a comparação do delta da excreção de sódio foi aplicada ANOVA com pos hoc de Bonferroni. O delta da excreção de sódio foi calculado mediante a subtração do valor desse exame obtido na V4 com aquele da V1b. A correlação entre os valores de PA (MRPA, casual e central) e com os níveis de sódio urinário foi realizada com o teste de Spearman. Considerou-se como significativos valores de p < 0,05.

## Resultados

Foram avaliados 55 participantes, 32 (58,2%) do sexo masculino, com a mediana da idade de 48 anos (IQ:39-54); 18 (32,7%) eram normotensos (GN), 15 (27,3%) pré-hipertensos (GPH) e 22 (40,0%) hipertensos (GH). Os grupos eram semelhantes em relação à idade e distribuição por sexo. Houve diferença entre os grupos com relação ao IMC (p = 0,03) (Tabela 1).

**Tabela 01:** – Caracterização da amostra segundo variáveis sociodemográficas e clínicas, Goiânia, Goiás, 2014, n = 55

Variáveis	Normotenso (n = 18)		Pré-hipertensos (n = 15)		Hipertenso (n = 22)		p*

	Mediana	IQ	Mediana	IQ	Mediana	IQ	
Idade	45,0	30-52	46,0	43-54	52,0	41-56	0,08
IMC	25,1	23,5-27,2	27,6	25,7-31,1	28,4	25,6-31,8	0,03
Sexo	N	%	N	%	N	%	p^†^
Masculino	09	50,0	11	73,3	12	54,5	0,424
Feminino	09	50,0	04	26,7	10	45,5	

*Teste de Kruskal-Wallis; ^†^ Teste de Fisher; valor de p significativo < 0,05; IMC: índice de massa corporal (kg/m^2^); IQ: intervalo interquatil. Fonte: os autores.

Não houve diferença nas medidas de pressão central e rigidez arterial entre as visitas V1B e V4 em nenhum dos grupos, entretanto houve uma tendência a redução dos valores centrais da PA tanto sistólica quanto diastólica em todos os grupos de V1B para a V4 (Tabela 2).

**Tabela 02:** – Comparação intra grupo (V1B e V4) das variáveis referentes a pressão arterial central, Goiás, 2014, n = 55

Variáveis	Normotenso (n = 18)		p	Pré-hipertensos (n = 15)		p	Hipertenso (n = 22)		p
		
	V1b	V4		V1b	V4		V1b	V4	
		
	Média ± DP	Média ± DP		Média ± DP	Média ± DP		Média ± DP	Média ± DP	
PASc	107,2 ± 9,2	103,4 ± 10,4	0,24	119,2 ± 8,5	115,0 ± 9,9	0,22	119,012,6	113,4 ± 9,0	0,10
PADc	73,34,7	70,6 ± 7,0	0,18	82,3 ± 8,9	74,7 ± 19,6	0,17	83,3 ± 11,6	78,5 ± 8,3	0,12
PPc	34,16,6	32,3 ± 7,5	0,45	36,9 ± 6,5	35,1 ± 4,9	0,41	35,6 ± 6,7	34,9 ± 6,0	0,70
AIx 75%	22,5 ± 15,3	21,8 ± 13,0	0,87	23,4 ± 10,4	19,7 ± 11,7	0,36	25,010,5	23,3 ± 10,5	0,59

DP: desvio padrão; *Teste T – Student para amostra pareada ou Teste Wilcoxon; PASc:pressão arterial sistólica central (mmHg); PADc: pressão arterial diastólica central (mmHg); PPc: pressão de pulso central (mmHg); ALx 75%: augmentation índex 75%. Fonte: os autores.

**Figura 2:**
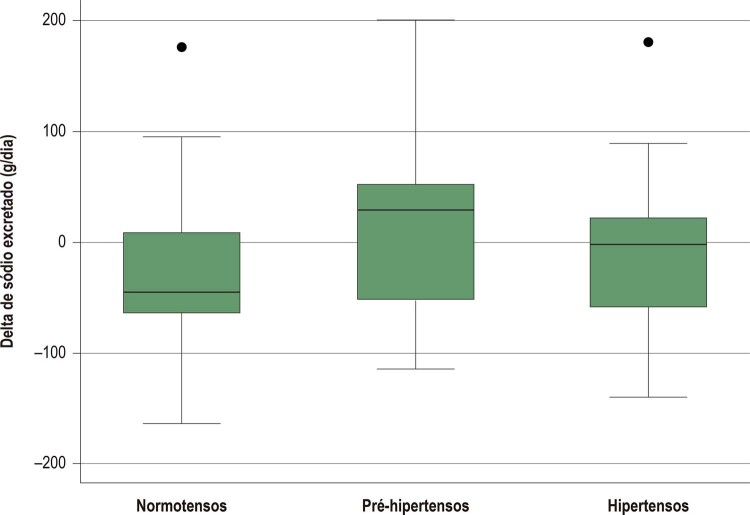
– *Comparação do delta de excreção de sódio entre os grupos normotensos, pré-hipertensos e hipertensos, Goiânia, 2014. ANOVA com pos hoc Bonferroni.*

Não houve diferença entre os grupos quanto ao delta da excreção de sódio (Figura 2).

Também não foram identificadas diferenças entre as medidas da V1B e V4 referentes a MRPA, pressão casual e sódio. Entretanto, houve uma tendência de redução da PAS e PAD casual e sódio urinário de V1B para a V4 nos grupos de normotensos e pré-hipertensos (Tabela 3).

**Tabela 03:** – Comparação intra grupo (V1B e V4) das variáveis referentes a MRPA, medida casual da PA, creatinina sérica e sódio urinário. Goiânia, Goiás, 2014, n = 55

Variáveis	Normotensos n = 18			Pré-hipertensos n = 15			Hipertensos n = 22		

	Média	DP	p	Média	DP	p	Média	DP	p
MRPA PAS V1b	115,7	9,7	0,87*	125,0	8,2	0,88*	128,6	10,3	0,63*
MRPA PAS V4	115,1	11,8		125,5	11,8		127,0	11,6	
MRPA PAD V1b	69,3	6,6	0,86*	76,1	7,7	0,72*	80,0	7,3	0,81*
MRPA PAD V4	69,7	7.2		77,3	10,8		79,5	8,0	
PAS casual V1B	116,3	10,6	0,44*	125,9	8,4	0,94*	128,5	11,1	0,18*
PAS casual V 4	113,8	9,3		126,1	7,5		124,2	10,1	
PAD casual V1B	71,1	7,4	0,58*	79,3	8,8	0,87*	81,4	8,5	0,10*
PAD casual V 4	69,8	7,2	0,44*	78,8	7,3		77,3	73,8	
Sódio V1B	163,2	71,7	0,20*	158,4	68,7	0,60*	156,8	52,7	0,63*
Sódio V4	135,2	58,1	0,20**	172,7	80,8	0,60**	147,3	75,9	0,63**

DP: desvio padrão; *Teste T – Student para amostra pareada; **Teste Wilcoxon; MRPAS: monitoramento residencial da pressão arterial sistólica (mmHg); MRPAD: monitoramento residencial da pressão arterial diastólica (mmHg); PAS casual: pressão arterial sistólica casual (mmHg); PAD casual: pressão arterial diastólica casual (mmHg); V 1b: visita 1b; V2: visita 2; V3: visita 3; V4: visita 4. Fonte: os autores.

O sódio urinário correlacionou-se com a PADc e periférica no grupo de hipertensos (Tabela 4).

**Tabela 4:** – Correlação entre as variáveis de pressão arterial com os valores de excreção de sódio de 24 horas na visita final, Goiânia, 2014

Variáveis	Normotensos n = 18		Pré-hipertensos n = 15		Hipertensos n = 22	

	R	p	R	p*	R	p
PASc x sódio V4	0,208	0,40	0,282	0,30	0,276	0,21
PADc x sódio V4	0,397	0,10	0,328	0,23	0,458	0,03*
PPc x sódio V4	-0,024	0,92	0,023	0,93	-0,174	0,43
AIx 75% x sódio V4	0,201	0,42	0,014	0,95	0,116	0,60
MRPA PAS x sódio V4	0,241	0,33	0,216	0,43	0,298	0,17
MRPA PAD x sódio V4	0,188	0,45	0,205	0,46	0,369	0,09
PAS casual x sódio V4	0,010	0,96	0,294	0,28	0,157	0,48
PAD casual x sódio V4	0,156	0,53	0,413	0,12	0,480	0,02*

Teste de Spearman; r: valor de rho; * ≤ 0,005; PASc: pressão arterial sistólica central (mmH; PADc: pressão arterial diastólica central (mmHg) (mmHg); PPc: pressão de pulso central (mmHg); ALx75%: augmentation índex 75%; MRPA: monitoramento residencial da pressão arterial; PAS casual: pressão arterial sistólica casual (mmHg); PAD casual: pressão arterial diastólica casual (mmHg); V1b: visita 1b; V2: visita 2; V3: Visita 3; V4: visita 4. Fonte: os autores

## Discussão

Em todas as metodologias utilizadas para a avaliação da PAS e PAD, não se observou alterações significativas com a redução gradual do sódio. Havia uma expectativa dos pesquisadores que os valores centrais da PA, por avaliarem o comportamento em artérias mais elásticas, pudesse apresentar uma sensibilidade maior na detecção de pequenas reduções dos níveis tensionais, o que também não ocorreu.

Dados da literatura associam a diminuição da ingestão de sal com redução da PA tanto em hipertensos quanto em normotensos e pré-hipertensos e descrevem maior sensibilidade da medida central da PA para detectar essas alterações. Entretanto boa parte dessas publicações interviu ou avaliou reduções principalmente no sal de alimentos industrializados ou o sal total ingerido.^18,19^

Em revisão sistemática, uma redução média de 4,4 g de sal/dia diminuiu a PA em normotensos em 2,4 mmHg na PAS e 1,0 mmHg na PAD e em hipertensos 5,4 mmHg na PAS e 2,8 mmHg na PAD. Isso indicou que, para cada grama reduzida por dia de sal, houve uma diminuição de 0,72 mmHg nos valores pressóricos de normotensos e 1,8 mmHg em hipertensos.^18^

A diminuição dos valores pressóricos implica em redução de eventos cardiovasculares, inclusive a mortalidade cardiovascular, fato que reforça a importância da adoção de medidas efetivas na redução do consumo de sal. Estudo realizado na Inglaterra, entre 2003 e 2011, avaliou a relação entre a redução da ingestão de sal total com a PA e a mortalidade por acidente vascular cerebral (AVC) e infarto agudo do miocárdio (IAM) e registrou que, a diminuição de 1,4g de sal diária levou a uma redução na PAS de 2,7 mmHg e na PAD de 1,1 mmHg. Desta forma, estimou-se que a redução de 2,7 na PAS mmHg provoca uma redução de 11% de AVC e de 6% de IAM. Isso corresponde a uma redução de mortalidade por AVC em 42% e IAM em 40%.^19^

No nosso estudo, as variáveis PPc e Alx75%, também não apresentaram significância estatística em suas reduções. Pelos motivos já descritos anteriormente, como essas variáveis se relacionam com resistência vascular e rigidez arterial, também havia a expectativa de reduções significativas, o que não ocorreu. Da mesma forma, acreditamos que uma intervenção em todo o conteúdo de sal ingerido poderia ter sido exitosa, conforme outras publicações demonstraram.^20^

Em estudo realizado com população hipertensa sul-africana, que avaliou a relação da ingestão de sal total a partir da excreção urinária de sódio 24 horas com hemodinâmica central, encontrou correlação com os parâmetros de rigidez arterial: PPc, AIx75%, PASc e pressão arterial média central (PAMC).^20^

Estudo realizado na China com hipertensos não tratados, avaliou a associação entre aumento na ingestão de sal, por meio da excreção urinária de 24 horas, com a PAC em três grupos. Grupo A: Excreção média de sódio de 76,9 mmol, Grupo B de 146,6 mmol e no Grupo C de 258,6 mmol, equivalentes respectivamente a 4,7g, 9,6 g e 15,8 g de ingestão de sal por dia. A média de excreção de sódio de todos os indivíduos foi de 166,6 mmol, equivalente a 10,1 g de ingestão de sal por dia. Houve piora progressiva nos parâmetros de rigidez arterial (PASc, PADc e AIx75%) do grupo A para o grupo C.^21^

Uma metanálise, que avaliou o efeito da redução na ingestão de sódio em relação a vários desfechos intermediários, dentre eles a PA, identificou que, após 03 semanas, já se observava reduções médias na PAS de 3,39 mmHg e de 1,54 mmHg da PAD. Essa redução foi maior em hipertensos (4,06 mmHg de PAS e 2,26 mmHg de PAD) que em normotensos (1,38 mmHg de PAS e 0,58 mmHg de PAD). Observou-se ainda que, comparando os grupos com ingestão diária de sal ≥ 2 g/dia versus < 2 g/dia, ou aqueles que reduziram em ≥ 1/3 do consumo diário de sal versus < 1/3 desse consumo, os primeiros sempre apresentaram maiores reduções nos valores da PA.^22^

Uma intervenção controlada na dieta, suplementando a ingestão de sal em 7,6 g por dia versus grupo placebo (sem suplementação), provocou aumento significativo nos valores centrais da PA, PASc em 8,5 mmHg, PADc em 3,6 mmHg e PPa em 4,8 mmHg.^23^

Fica claro, portanto, que estratégias voltadas para redução na quantidade de sal ingerida em alimentos industrializados ou que atuem no sal total consumido são eficazes como medidas não medicamentosas na prevenção e no tratamento da hipertensão arterial.

A nossa opção em atuar apenas no sal de adição teve como objetivo avaliar se essa estratégia, comum nas orientações dos profissionais de saúde, e por um curto período de tempo, seria efetiva em reduzir a PA. Vale ressaltar que a recomendação da OMS é para uma redução para 5 g ao dia no total de sal da dieta, eficaz em reduzir os níveis pressóricos.^15^

É possível que uma intervenção no sal de adição em um número maior de refeições assim como, por um período mais longo, apresente maior eficácia que a encontrada em nossos resultados. Uma metanálise de estudos que avaliaram intervenções para redução na ingestão de sal, demonstrou que, um período de até cinco semanas para indivíduos hipertensos e até quatro semanas para normotensos é insuficiente para obter o efeito máximo da redução da PA.^18^ No nosso estudo, a opção de quatro semanas de intervalo entre cada nível de redução do sal de adição teve o propósito de manter uma boa adesão à intervenção proposta.

Baseados nas evidências científicas, países europeus têm realizado recomendações populacionais para manter o consumo de sal total abaixo de 5g/dia. No Reino Unido e na Finlândia definiu-se como política de governo, atingir uma meta de redução no consumo de sal para menos de 3 g/dia até o ano de 2025.^24^

Essas medidas governamentais são fundamentais na prevenção de milhões de doenças relacionadas aos danos desencadeados pelo excesso de sódio na dieta. A redução no consumo de sódio em até 2.300 mg/dia pode prevenir 11 milhões de casos de HAS e economizar bilhões de dólares em gastos na área da saúde.^24^ Uma metanálise demonstrou que, a redução acentuada do consumo de sal, limitando a ingestão para 3g sal/dia, foi eficaz na prevenção das DCV. Grande parte dessa prevenção decorre da redução nos níveis pressóricos, que ocorre tanto em hipertensos quanto em pré-hipertensos e normotensos.^18^

Outra estratégia interessante pode ser a substituição do sal de adição pelo sal light. Estudo randomizado com hipertensos não controlados, identificou redução nos valores de PA e na excreção urinária de sódio no grupo que recebeu 3 g/dia de sal light em detrimento do que recebeu sal de adição.^25^

Todas essas estratégias são importantes, mas, de forma isolada, são insuficientes. Os nossos resultados reforçam a necessidade de atuar de forma incisiva na redução do teor de sal principalmente nos alimentos industrializados, que habitualmente contém elevadas quantidades de sódio. Os alimentos processados, presentes em grandes quantidades na dieta pós-moderna, são responsáveis pela maior parte do sódio consumido.^26^

Também é importante a adoção de informações mais claras e objetivas, nos diversos produtos, sobre o teor de sal, de forma que os consumidores possam, de forma consciente, adotar mudanças ou adequações nos seus hábitos alimentares.^27^

Outro aspecto interessante é que a dosagem do sódio urinário, apesar de ser o padrão ouro, apresenta sensibilidade de 86% na detecção do sódio excretado e a intervenção no sal de adição representa uma ação em apenas 15% do sal total ingerido, ou seja, a sensibilidade do método em detectar alterações na excreção de sódio nesse modelo de intervenção tende a ser baixa, exatamente o que acreditamos ter ocorrido em nossa amostra. Além disso, há que se considerar que a adesão a esse tipo de intervenção sofre variações de indivíduo para indivíduo e pode ser baixa. Na nossa amostra não observamos redução do sódio excretado em nenhum dos grupos.

Outro aspecto a ser considerado é que não há como assegurar a correta realização do exame pois não verificamos se o armazenamento e a coleta urinária durante as 24 horas foram adequados. De qualquer modo esse é o método utilizado por diversos pesquisadores no Brasil^28^ e no mundo.^29,30^

A metanálise que avaliou estudos que realizaram intervenções de quatro semanas a três anos com redução moderada da ingestão de sal para avaliar os efeitos na excreção de sódio urinário de 24 horas e na PA identificou que a redução média do consumo de sal foi 4,4 g/dia e produziu uma redução de 5,4 mmHg na PAS e 2,4 mmHg na PAD em hipertensos e, 2,4 mmHg na PAS e 1,0 mmHg na PAD em normotensos. Dessa forma, uma redução moderada na ingestão de sal por períodos mais longos se mostrou eficaz na redução dos níveis tensionais.^18^

Uma das limitações do nosso estudo foi a dificuldade de controlar, de forma efetiva, que os participantes realizassem pelo menos quatro refeições principais em suas residências semanalmente e que utilizassem somente o sal de adição entregue pelo estudo no preparo dos alimentos. Também não houve controle das refeições realizadas fora do domicílio. A estratégia que utilizamos foi envolver toda a família na redução do consumo de sal de adição e reforçar a orientação sobre alimentos com alto teor de sódio oferecidos em restaurantes, assim como a escolha de alimentos com baixo teor de sódio.

## Conclusões

O modelo de intervenção proposto com redução gradativa do sal de adição de 6 para 4 g/dia por um período de 13 semanas, não foi capaz de demonstrar redução na quantidade de sal excretado na urina de 24 horas, entretanto, o sódio final excretado apresentou correlação positiva e moderada com a PADc e PAD casual no grupo de hipertensos.
