# Supplementary material for: Efeito da Redução do Sal de Adição sobre a Pressão Arterial Central e Periférica
Source: Arq Bras Cardiol. 2020 Apr 6;114(3):554–61. [Article in Portuguese] doi: 10.36660/abc.20180426 (PMC7792722; doi:10.36660/abc.20180426)
Supplement: Translation

# Translation: Effects of added salt reduction on central and peripheral blood pressure

**Authors:** Ana Carolina Arantes, Ana Luiza Lima Sousa, Priscila Valverde de O. Vitorino, Paulo Cesar B. Veiga Jardim, Thiago de Souza Veiga Jardim, Jeeziane Marcelino Rezende, Ellen de Souza Lelis, Rafaela Bernardes Rodrigues, Antonio Coca, Weimar Kunz Sebba Barroso

**Affiliations:** 1 BrazilUniversidade Federal de Goiás - Faculdade de Medicina - Programa de Pós-Graduação em Ciências da Saúde, Goiânia, GO – Brazil; 2 BrazilUniversidade Federal de Goiás - Liga de Hipertensão Arterial, Goiânia, GO – Brazil; 3 BrazilPontificia Universidade Católica de Goiás - Escola de Ciências Sociais e da Saúde - Mestrado em Atenção à Saúde, Goiânia, Goiás – Brazil; 4 SpainUniversitat de Barcelona, Barcelona – Spain

**Keywords:** Cardiovascular Diseases, Arterial Pressure, Prehypertension, Hypertension, Sodium Chloride, Diet, Sodium-Restricted, Health Policies

## Abstract

**Background:**

Although the effects of salt intake reduction on casual blood pressure have been extensively studied in hypertensive individuals, data on reductions of added salt on arterial stiffness in both normotensive and prehypertensive subjects are scarce.

**Objective:**

To evaluate the effects of progressive reduction in added salt intake (from 6 grams to 4 grams per day) on peripheral and central blood pressure and arterial stiffness in normotensive, prehypertensive and hypertensive individuals.

**Methods:**

This was a single-blinded clinical trial with 13 weeks of follow-up. Normotensive (≤ 130/85 mmHg), prehypertensive (≥ 130 e < 139/≥ 85 e < 90 mmHg) and stage 1 hypertensive individuals (< 139/≥ 85 and < 90 mmHg) were assessed. Casual blood pressure measurements and ambulatory blood pressure monitoring were performed using the automated OMRON 705CP device, and central blood pressure was measured using the Sphygmocor^®^. Twenty-four-hour urinary sodium excretion and the amounts of added salt consumed were measured. Statistically significance level was set at p < 0.05 for all analysis.

**Results:**

A total of 55 participants (18 normotensive, 15 prehypertensive and 22 hypertensive), median age 48 years (IQR:39-54) were studied. The groups were not different in age or sex. No difference was observed in blood pressure or sodium excretion levels before and after the intervention. No significant changes in arterial stiffness parameters were observed.

**Conclusion:**

The progressive reduction in added salt intake during a period of 13 weeks did not cause significant reductions in peripheral and central blood pressure. (Arq Bras Cardiol. 2020; 114(3):554-561)

## Introduction

Systemic arterial hypertension is one of the most prevalent cardiovascular risk factors, affecting nearly 970 million people in the world. It is the (direct or indirect) cause of more than nine million deaths every year,^1^ accounting for 62% of the cases of cardiovascular diseases (CVD) and 49% of ischemic heart disease.^2^ Prehypertension (PH) is also associated with increased incidence of CVD.^3,4^

Compared with other methods of blood pressure (BP) measurement, casual BP measurement is inferior in predicting cardiovascular risk and shows lower diagnostic accuracy.^5,6^ Ambulatory blood pressure monitoring (ABPM) has high diagnostic accuracy and excellent cost-benefit relationship.^7,8^ Central blood pressure (CBP) provides information of more elastic, central arteries; it has lower values compared with casual pressure and is better associated with lesions in target organs. Therefore, CPB is the best predictor of cardiovascular events,^8^ in addition to allowing the analysis of arterial stiffness and vascular resistance parameters.^9-12^

Although the etiology of increased BP is multifactorial, excessive salt intake is a common and important factor. It causes elevations of BP levels and cardiovascular complications. Therefore, salt restriction is an important strategy for prevention and control of systemic arterial hypertension and CVD.^13,14^

Mean daily amount of salt intake recommended is 5 g, or 2 g of sodium. However, Brazilians eat on average up to 12 g/day, i.e., more than the daily amount recommended.^15^ Government policies of many countries have been implemented to reduce salt intake by 30% by the year of 2025 with the aim to reduce BP values in the population.^16^

Assessment of salt intake, interventions for its reduction, and the use of instruments capable of identifying this reduction are important strategies in primary prevention of CVD.

Therefore, this study evaluated the effect of reducing the intake of added salt on central and peripheral BP in normotensive, prehypertensive and hypertensive individuals after 13 weeks of follow-up.

## Methods

This is a substudy of the phase II, single-blinded, controlled clinical trial with different amounts of added salt in individuals grouped by BP levels. For the initial sample, a total of 1,000 workers were recruited at a Brazilian public university. A questionnaire on dietary habits was administered, and anthropometric and casual BP measurements were performed. Of the subjects recruited, 678 agreed to participate (Figure 1).

**Figure 01:** – *Flowchart of the phase II clinical trial.*

The study was approved by the ethics committee (CAEE: 00790712.3.0000.5078) and all participants signed an informed consent form.

The study population was a convenience sample and was composed of men and women aged between 20 and 60 years. All participants had at least four main meals (lunch and/or dinner) a week at home.

Individuals with casual BP ≥ 160/100 mmHg, diabetes, history of chronic disease and hypertension taking two or more antihypertensive were excluded.

Participation of the study consisted of five visits with an interval of 30 ± 7 days between them. The first visit was divided into two parts, Visit 1A (V1A) and Visit 1B (V1B). In all visits, measurements of casual BP, CBP, ABPM and BMI were performed, and request for urinalysis, and 24-hour urine creatinine, sodium and potassium was made. In V1A, in addition to these procedures, participants also signed the informed consent form, were evaluated for eligibility criteria and a request for serum creatinine was made.

In V1B, participants were grouped according to mean casual BP, in normotensive (NG) (BP < 130/85 mmHg), prehypertensive (PHG) (BP ≥ 130 < 140/≥85 < 90 mmHg) and stage 1 hypertensive (HG) (BP ≥ 140 and < 160/≥ 90 and < 100 mmHg) not using antihypertensive medication.^1^

For casual BP, three measurements were taken with a minimum interval of one minute between them. When a difference greater than 4 mmHg was found between the measurements, further measures were taken until the differences between them were smaller.

Both casual BP and ABPM were measured using a semiautomated device (OMRON, model HEM-711 ACINT), with a cuff size according to the arm circumference. BP was measured in the sitting position after a resting period of five minutes, in a calm environment in the arm with the highest BP value.^1^

ABPM was performed according to the II Brazilian Guidelines for Ambulatory Blood Pressure Monitoring.^17^ In each visit, the ABPM device was given to each participant, who was instructed to obtain BP measures following specific protocol, to write down the values in a proper document and to return the device at the next visit.

CBP measurements were obtained by applanation tonometry, using a calibrated and validated device (Sphygmocor®).^12^ Each patient refrained from alcohol, coffee and tobacco use for some hours before the exam, which was performed with empty bladder, after a five-minute rest. The variables analyzed from CBP were central systolic blood pressure (cSBP), central diastolic blood pressure (cDBP), central pulse pressure (cPP) and augmentation index (AIx).

Collection of the 24-hour urine sample was conducted following the information contained in an explanatory leaflet. The 24-hour urine test was performed at the laboratory of the Federal University of Goias, and an ion-selective membrane was used to quantify urinary sodium at baseline, before the intervention and in the intervals between the visits (total of four collections).

During V1B, the NG, PHG and HG received the same instructions regarding the amount of salt intake (6g/day). In visits 2 (V2) and 3 (V3), 5 g/day and 4 g/day of salt, respectively, were given to each participant. Interval between the visits was of 30 ± 7 days.

The amount of salt given to each participant was estimated based on the number of people living in the residence and the meals (lunch and dinner) prepared. The salt was delivered properly packed, without weight identification. Also, an additional 10% of salt was given to each participant, to be used in exceptional cases (e.g. visitors at home).

In the return visits (V2, V3 and visit 4, V4), all packages of salt were collected and other packages containing the amount of salt planned for the subsequent period were given. In all visits, it was emphasized to participants the importance of cardiovascular health and of a low-sodium diet, and that the amount of added salt consumed by participants should be limited to that established in the study protocol.

The salt packages (empty or full) returned were weighed and used for assessment of adherence to the protocol, which was also evaluated by 24-hour urinary excretion.

### Statistical analysis

Statistical analysis was performed using Stata, version 12. An intention-to-treat analysis was used, and for those who dropped out the study before V4, the data of the last visit were considered for analysis. Continuous variables with normal distribution were presented as mean and standard deviation, and those with a non-normal distribution were presented as median and interquartile range. Categorical variables were presented as absolute and relative frequency. Normal distribution of data was tested using the Shapiro-Wilk test.

Between-group comparisons in V1A were made using the Kruskal-Wallis test and the Fisher’s exact test. Within-group comparisons before (V1B) and after (V4) intervention were performed by Wilcoxon test or the paired Student’s t-test. Comparison of delta sodium excretion was made by ANOVA followed by Bonferroni post hoc test. Delta sodium excretion was calculated by subtracting sodium excretion at V4 from that obtained in V1B. Correlation between BP (ABPM, and casual and central BP) and the levels of urinary sodium was performed by Spearman’s test. A p < 0.05 was considered statistically significant.

## Results

Fifty-five individuals participated in the study, 32 (58.2%) were male, median age of 48 years (IQ:39-54). Eighteen (32.7%), 15 (27.3%) and 22 (40.0%) individuals were included in the NG, PHG and HG, respectively. There was no difference in age and sex between the groups, but a significant difference was observed in BMI (p = 0.03) (Table 1).

**Table 01:** – Sociodemographic and clinical characteristics of the study sample (n = 55), Goiânia, Brazil, 2014

Variables	Normotensive (n = 18)		Prehypertensive (n = 15)		Hypertensive (n = 22)		p*

	Median	IQ	Median	IQ	Median	IQ	
Age	45.0	30-52	46.0	43-54	52.0	41-56	0.08
BMI	25.1	23.5-27.2	27.6	25.7-31.1	28.4	25.6-31.8	0.03
Sex	N	%	N	%	N	%	p^†^
Male	09	50.0	11	73.3	12	54.5	0.424
Female	09	50.0	04	26.7	10	45.5	

*Kruskal-Wallis test; ^† ^Fisher’s exact test; p-value < 0.05 was considered significant; BMI: body mass index (kg/m^2^); IQR: interquartile range.

No difference was observed in CBP and AS between V1 and V4 in any of the groups. However, there was a trend of reduction in both cSBP and cDBP from V1 to V4 in all groups (Table 2). There was no difference in delta sodium excretion between the groups (Figure 2).

**Table 02:** – Within-group comparisons (Visits 1B and 4) of central blood pressure parameters (n = 55)

Variables	Normotensive group (n = 18)		p	Prehypertensive group (n = 15)		p	Hypertensive group (n = 22)		p
		
	V1b	V4		V1b	V4		V1b	V4	
		
	Mean ± SD	Mean ± SD		Mean ± SD	Mean ± SD		Mean± SD	Mean ± SD	
cSBP	107.2 ± 9.2	103.4 ± 10.4	0.24	119.2 ± 8.5	115.0 ± 9.9	0.22	119.0 ± 12.6	113.4 ± 9.0	0.10
cDBP	73.3 ± 4.7	70.6 ± 7.0	0.18	82.3 ± 8.9	74.7 ± 19.6	0.17	83.3 ± 11.6	78.5 ± 8.3	0.12
cPP	34.1 ± 6.6	32.3 ± 7.5	0.45	36.9 ± 6.5	35.1 ± 4.9	0.41	35.6 ± 6.7	34.9 ± 6.0	0.70
AIx 75%	22.5 ± 15.3	21.8 ± 13.0	0.87	23.4 ± 10.4	19.7 ± 11.7	0.36	25.0 ± 10.5	23.3 ± 10.5	0.59

SD: standard deviation; *paired Student’s t-test or Wilcoxon test; cSBP: central systolic blood pressure (mmHg); cDBP: central diastolic blood pressure (mmHg); cPP: central pulse pressure (mmHg); ALx 75%: augmentation index 75%.

**Figure 2:** –* Comparison of delta sodium excretion between normotensive, prehypertensive and hypertensive groups; ANOVA with Bonferroni post-hoc test.*

In addition, no differences were found in ABPM, casual BP or urinary sodium from V1B to V4 in NG and PHG (Table 3).

**Table 03:** – Within-group comparison (Visits 1B and V4) of ambulatory blood pressure monitoring, casual blood pressure, serum creatinine and urinary sodium (n = 55)

Variables	Normotensive group n = 18			Prehypertensive group n = 15			Hypertensive group n = 22		

	Mean	SD	p	Mean	SD	p	Mean	SD	p
ASBPM V1b	115.7	9.7	0.87*	125.0	8.2	0.88*	128.6	10.3	0.63*
ASBPM SBP V4	115.1	11.8		125.5	11.8		127.0	11.6	
ADBPM V1b	69.3	6.6	0.86*	76.1	7.7	0.72*	80.0	7.3	0.81*
ADBPM V4	69.7	7.2		77.3	10.8		79.5	8.0	
casual SBP V1B	116.3	10.6	0.44*	125.9	8.4	0.94*	128.5	11.1	0.18*
casual SBP V4	113.8	9.3		126.1	7.5		124.2	10.1	
Casual DBP V1B	71.1	7.4	0.58*	79.3	8.8	0.87*	81.4	8.5	0.10*
Casual DBP V 4	69.8	7.2	0.44*	78.8	7.3		77.3	73.8	
Sodium V1B	163.2	71.7	0.20*	158.4	68.7	0.60*	156.8	52.7	0.63*
Sodium V4	135.2	58.1	0.20**	172.7	80.8	0.60**	147.3	75.9	0.63**

SD: standard deviation; *paired Student’s t-test; **Wilcoxon test; ASBPM: ambulatory systolic blood pressure monitoring (mmHg); ADBPM: ambulatory diastolic blood pressure monitoring (mmHg); SBP: systolic blood pressure (mmHg); DBP: diastolic blood pressure (mmHg); V1b: visit 1b; V2: visit 2; V3: visit 3; V4: visit 4.

Urinary sodium correlated with CPB and peripheral BP in the HG (Table 4).

**Table 4:** – Correlation of blood pressure parameters with 24-hour urinary sodium excretion, n = 55

Variables	Normotensive group n = 18		Prehypertensive group n = 15		Hypertensive group n = 22	

	r	p	r	p*	r	p
cSBP x sodium V4	0,208	0,40	0,282	0,30	0,276	0,21
cDBP x sodium V4	0,397	0,10	0,328	0,23	0,458	0,03*
PPc x sodium V4	-0,024	0,92	0,023	0,93	-0,174	0,43
AIx 75% x sodium V4	0,201	0,42	0,014	0,95	0,116	0,60
ASBPM x sodium V4	0,241	0,33	0,216	0,43	0,298	0,17
ADBPM x sodium V4	0,188	0,45	0,205	0,46	0,369	0,09
casual SBP x sodium V4	0,010	0,96	0,294	0,28	0,157	0,48
Casual DBP x sodium V4	0,156	0,53	0,413	0,12	0,480	0,02*

Spearman test; r: rho value; * ≤ 0.005; ASBPM: ambulatory systolic blood pressure monitoring (mmHg); ADBPM: ambulatory diastolic blood pressure monitoring (mmHg); SBP: systolic blood pressure (mmHg); DBP: diastolic blood pressure (mmHg); V4: visit 4.

## Discussion

Based on the methods used for SBP and DBP assessment in the study, the progressive reduction of salt intake was not associated with significant changes in SBP. Also, the authors expected to find a higher sensitivity of CBP in detecting small changes in tension, since this parameter reflects the behavior of more elastic arteries, which did not occur.

Data from the literature have associated the reduction in salt intake with a reduction in BP in hypertensive, normotensive and prehypertensive individuals and have shown a higher sensitivity of CBP to detect these changes. However, a large part of these studies was based on interventions or evaluated reductions in the consumption of salt in packaged food and total intake.^18,19^

In a systematic review, a mean reduction of 4.4 g/day was associated with a reduction by 2.4mmHg in SBP and 1.0 mmHg in DBP in normotensive subjects, and by 5.4 mmHg in SBP and 2.8 mmHg in DBP in hypertensive subjects. These findings indicated a reduction of 0.72 mmHg ad 1.8 mmHg in BP levels in normotensive and hypertensive individuals, respectively, for each gram of salt reduction daily.^18^

Improvements in BP levels lead to lower cardiovascular events, including cardiovascular mortality, which reinforces the importance of adopting effective measures to reduce salt consumption. A study conducted in England between 2003 and 2011 evaluated the relationship of reductions in total salt intake with BP and mortality for stroke and acute myocardial infarction and showed a reduction by 2.7 mmHg in SBP and 1.1 mmHg in DBP. Therefore, a fall of BP of 2.7 mmHg led to a decrease in mortality for stroke by 42% and acute myocardial infarction by 40%.^19^

In our study, the variables cPP and AIx75% did not show statistically significant reductions, which is in contrast to what the authors expected, since these variables are also related to vascular resistance and arterial stiffness. Again, in our opinion, this may be achieved by an intervention aimed at reducing total salt intake, as previous studies have already demonstrated.^20^

In a study conducted with South African hypertensive individuals, the authors evaluated the relationship between salt intake (assessed by 24-hour urinary sodium) and central dynamics and found a correlation with arterial stiffness parameters – cPP, AIx75%, cSBP and central mean arterial pressure.^20^

A study conducted in China evaluated the association between salt intake (24-hour urinary excretion) with CBP in three groups of untreated hypertensive patients divided into three groups according to mean urinary sodium excretion – group A, 76.9 mmol, group B, 146.6 mmol and group C 258.6 mmol, corresponding respectively to 4.7 g, 9.6 g and 15.8 g of salt per day. The mean 24-h urinary sodium of all patients was 166.6 mmol, or 10.1g of salt. Parameters of arterial stiffness (cSBP, cDBP and AIx75%) worsened from group A to group C.^21^

A meta-analysis evaluating the effect of salt intake reduction on intermediate outcomes, including BP, detected mean BP reductions of 3.39 mmHg in SBP and 1.54 mmHg in DBP. Such effect was greater in hypertensive (4.06 mmHg in SBP and 2.26 mmHg in DBP) than normotensive individuals (1.38 mmHg in SBP and 0.58 mmHg in DBP). In addition, greater reductions in BP was observed in individuals with sodium intake < 2 g/day versus ≥ 2 g/day, and in those with a reduction in daily salt intake ≥ 1/3 versus < 1/3.^22^

A controlled dietary intervention consisting of 7.6 g/day sodium supplementation versus placebo (no supplementation) caused a significant increase in CBP measurements – 8.5 mmHg in SBP, 3.6 mmHg in cDBP and 4.8 mmHg in PPa.^23^

It is therefore clear that strategies towards reductions in salt intake (salt in packaged foods or total salt consumption) are an effective nonpharmacological approach for the prevention and treatment of hypertension.

Since reducing the amount of added salt in the diet is commonly recommended by healthcare professionals, we decided to investigate whether such strategy, adopted for a short period of time, would be effective in reducing BP levels. It is worth pointing out that the World Health Organization recommends the reduction in salt intake to less than 5 grams per day to reduce BP.^15^

It is possible that an intervention towards lowering added salt intake in more meals and for a longer period would lead to more effective results than those obtained in this study. A meta-analysis of studies on interventions of salt intake reduction showed that reductions in salt intake for up to five weeks in hypertensive individuals and for up to four weeks in normotensive individuals are ineffective to cause significant falls in BP.^18^ In our study, intervals between the different levels of salt reduction were of four weeks, aiming to achieve good adherence to the intervention proposed.

Based on scientific evidence, European countries have established population-wide recommendations to lower salt intake to less than 5 grams per day. In the United Kingdom and Finland, there are government policies focusing on reducing salt intake to less than 3 grams per day by the year of 2025.^24^

These governmental measures are crucial for preventing many diseases related to excessive salt intake. Lowering salt intake to up to 2,300mg per day could prevent 11 million cases of systemic arterial hypertension and save billions of dollars in health care costs.^24^ A meta-analysis showed that a drastic reduction in salt intake (up to 3g/day) was effective in preventing CVD. A major part of this prevention is explained by reductions of BP that occur in both hypertensive and prehypertensive individuals.^18^

Another interesting strategy may be the replacement of conventional salt with low-sodium salt. A randomized trial with patients with uncontrolled hypertension showed reductions in BP and urinary sodium in the group of individuals that received 3 grams of light salt compared with the group that received regular salt.^25^

All these strategies are important, but ineffective if used alone. Our results reinforce the need to sharply reduce the amount of salt intake, especially through packaged foods that usually contain great amounts of sodium. Processed foods are very present in post-modern society and the main sources of salt in the diet.^26^

It is also important the use of clear and objective information about salt content in packaged foods, so that consumers can deliberately change or make adaptations in their habitual diet.^27^

Also, although quantification of urinary sodium is the gold standard method to estimate sodium intake, it has a sensitivity of 86% in detecting urinary sodium excretion. Considering that interventions towards lowering added salt affect only 15% of total salt intake, the sensitivity of the method to detect changes in sodium excretion in these interventions is probably low, as may have occurred in our study. Besides, adherence to interventions like this varies between individuals and may be low. In our sample, we did not detect significant reductions in urinary sodium excretion in any of participants.

Another factor to be considered is that we cannot assure that the 24-hour urinary excretion test was performed correctly, since we did not verify how urine sample was collected and stored. However, this method has been used by different researchers in Brazil^28^ and in the world.^29,30^

The meta-analysis of trials with a modest reduction in salt intake and duration of four weeks to three years evaluating the effects on 24-hour urinary sodium excretion and BP showed that a reduction of 4.4g per day of salt was associated with a fall in SBP of 5.4 mmHg in normotensive individuals. Therefore, a moderate reduction in salt intake for longer periods was effective in reducing BP levels.^18^

One of the limitations of our study was the difficulty in ensuring that participants had at least four main meals at home per week and that the salt added during food preparation was only that received during the study. Out-of-home meals were not controlled also. The strategy used was to involve the whole family in lowering the amounts of added salt and to emphasize the importance of identifying high-sodium foods in restaurant and of choosing low-sodium foods.

## Conclusions

The intervention proposed, to gradually reduce the amount of added salt from 6 grams to 4 grams per day for 13 weeks, did not show significant reductions in the 24-hour urinary sodium excretion. However, the amount of sodium excretion showed a positive, moderate correlation with CBP and casual DBP in the HG.
